# Tendon Suture

**Published:** 1918

**Authors:** 


***Tendon Suture.*** By Torr Wagner Harmer. Abs. from the *Boston Medical and Surgical Journal.* Dec. 6, 1917. Vol. CLXXVII, No. 23, p. 808.

The author recommends a technique for tendon suture which he finds is not generally familiar. He describes it as follows:

The stitch is of silk and consists in overcasting the lateral margins of both ends of the divided tendon. The overcasting starts about the width of the tendon, or a little more, back from the point of division, and comprises several whippings about the side of the tendon down to the line of division, each loop including somewhat less than one-quarter of the circumference of the tendon. When a tendon is ready to be brought together, each end then carries two stitches and each stitch two ends. The two parts of the tendon are now brought together and the two suture ends nearest the line of division on one side are tied. The two pairs of stitch-ends on the other side are then similarly tied... The long ends tied along the side of the tendon seem to serve as lateral splints. The objection that they occasion too much suture material in the wound is refuted by the fact that the author has never seen any harm from them.

A dry wound before closing is desirable...

Care is taken in closing the wound to approximate the fascia and skin separately. *No splint is used.* Active motion is started as soon as the patient has recovered from the anesthetic. This will never be too energetically done on account of the pain which it causes.

The author gives in detail a case in which 24 tendons ends in the wrist of a five-year-old boy were approximated and sutured with almost perfect functional results.

